# Pluripotent stem cell differentiation as an emerging model to study human prostate development

**DOI:** 10.1186/s13287-020-01801-9

**Published:** 2020-07-16

**Authors:** Yangyang Yu, Wei Jiang

**Affiliations:** 1 Department of Biological Repositories, Frontier Science Center for Immunology and Metabolism, Medical Research Institute, Zhongnan Hospital of Wuhan University, Wuhan University, 116 East-Lake Road, District of Wuchang, Wuhan, 430071 Hubei Province China; 2 Hubei Provincial Key Laboratory of Developmentally Originated Disease, Wuhan, 430071 China; 3 Human Genetics Resource Preservation Center of Wuhan University, Wuhan, 430071 China

**Keywords:** Prostatic differentiation, Prostate development, Organoid, Pluripotent stem cell

## Abstract

Prostate development is a complex process, and knowledge about this process is increasingly required for both basic developmental biology studies and clinical prostate cancer research, as prostate tumorigenesis can be regarded as the restoration of development in the adult prostate. Using rodent animal models, scientists have revealed that the development of the prostate is mainly mediated by androgen receptor (AR) signaling and that some other signaling pathways also play indispensable roles. However, there are still many unknowns in human prostate biology, mainly due to the limited availability of proper fetal materials. Here, we first briefly review prostate development with a focus on the AR, WNT, and BMP signaling pathways is necessary for prostate budding/BMP signaling pathways. Based on the current progress in in vitro prostatic differentiation and organoid techniques, we propose human pluripotent stem cells as an emerging model to study human prostate development.

The prostate originates from the urogenital sinus (UGS), and cell lineage analysis demonstrates that the UGS is derived from the endoderm and gives rise to the entire urethra [1]. The UGS is partitioned into two parts, the urogenital sinus epithelium (UGE) and the surrounding urogenital sinus mesenchyme (UGM). Recombinant tissue experiments indicate that the interaction between the UGM and the UGE is sufficient to generate a well-developed prostate [2, 3]. It was known very early that the development of the prostate is under the control of androgens, which are secreted from the testes [4]. The fetal testes start to produce testosterone at approximately 9 weeks of gestation in humans and at E13–14 in mice, and testosterone is further converted into dihydrotestosterone, a more effective androgen, by 5α-reductase [5]. The androgen receptor (AR) pathway plays an extremely important role in the induction of the prostate [6].

Epithelial prostatic budding occurs at approximately 9–10 weeks of gestation in humans [7] and at E17.5 in mice [8] (Fig. 1). FOXA1, an important pioneer gene in endoderm-derived epithelial cells, is highly expressed in prostate epithelial cells [9]. FOXA1 can interact with closed chromatin and loosen nucleosomes as a pioneer factor, therefore allowing AR to bind to the DNA [10]. More importantly, it has been demonstrated that FOXA1 mutations disturb prostate differentiation [11]. In addition to AR, NKX3-1 is indispensable in prostate specification. NKX3-1 starts to be expressed 2 days prior to prostatic budding, suggesting that NKX3-1 may define the initiation of prostate organogenesis. Moreover, NKX3-1 uniquely marks the prostate epithelium and is not detected in other tissues of the male urogenital system. Functionally, a defect in NKX3-1 alters prostate development in mice [12]. In addition, the combination of AR, FOXA1, and NKX3-1 is identified as a driver for prostate organogenesis by a computational system approach named the Master Regulator Inference algorithm [13]. Moreover, these master regulators are used to reprogram induced epithelial cells derived from mouse fibroblasts into prostate tissue. These reprogrammed prostate-like cells exhibited appropriate histological and molecular properties of the prostate after grafting into a mouse model [13].

**Fig. 1:**
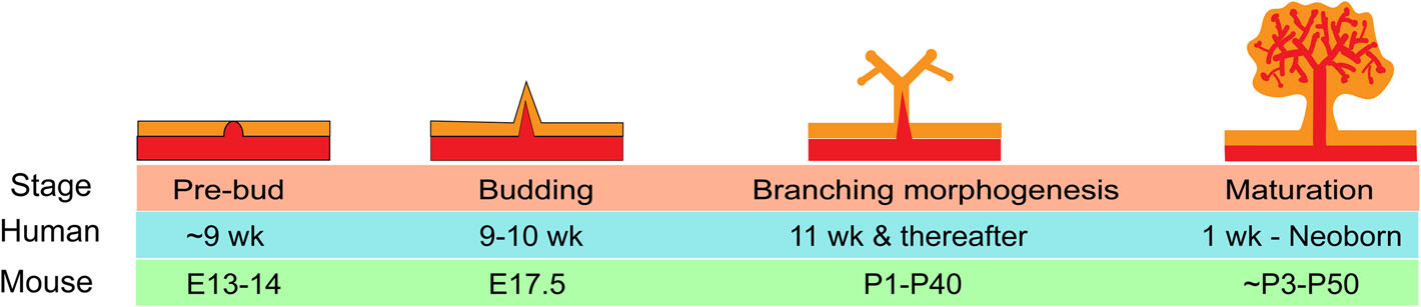
Major events during prostatic development and the respective developmental time in the mouse and human. Prostate originates from the progenitors within UGS, which goes through budding and branching morphogenesis, and eventually matures to the prostatic epithelium. wk, weeks after gestation; E, embryonic day; P, postnatal day

The next key events after prostatic budding are ductal outgrowth and branching morphogenesis. Budding starts during late fetal development, and the most prominent bud outgrowth occurs during the first two postnatal weeks in rodents [14]. The elongation and branching morphogenesis of human prostatic buds starts at 11 weeks of gestation and peaks at 16 weeks of gestation. In contrast to that in rodents, most of the prostate epithelium in humans is in the form of canalized ducts undergoing secretory cytodifferentiation before birth [15]. The theory of branching and annihilating random walks (BARWs) describes a process whereby the active tips elongate in all directions to randomly form ducts; ducts can branch through stochastic tip bifurcation at any moment or terminate when the tips meet with an existing duct [16]. The duct network can greatly maximize the exchange surface between the prostate epithelium and lumen. This model explains duct network heterogeneity and its spatiotemporal pattern. The FGF10/FGFR2IIIB interaction is reported to upregulate the expression of SHH and BMP7, which together contribute to duct branching morphogenesis. Additionally, SHH can downregulate FGF10 expression as a negative feedback loop and upregulate BMP4 expression, which controls duct elongation [17]. In addition, PI3K/mTOR signaling is required for prostate epithelial invasion and therefore regulates prostatic branching morphogenesis [18].

## AR/WNT/BMP signaling pathways in prostate development

AR is the most studied signal in both prostate development and prostate cancer. AR is recognized as a nuclear receptor but only enters the nucleus upon binding with androgen hormones and then exerts its function as a transcription factor [19]. Immediately after sex determination, dihydrotestosterone converted from testosterone by 5α-reductase binds to AR to activate the expression of NKX3-1, FOXA1, PSA, and other prostatic genes, therefore promoting prostate budding, branching morphogenesis, and maturation [20]. Testicular feminization mutant (Tfm) mice carrying a natural defect in the AR locus show an absence of prostatic buds, indicating the essential role of AR [21]. Conditional deletion of AR in both stromal fibroblasts and smooth muscle cells can impair branching morphogenesis in a mouse model [22], suggesting an additional critical role in subsequent ductal branching morphogenesis. In addition, AR and circulating androgens are both abundant in the UGS of male mice, while only the level of circulating androgens is quite low in females, which suggests that androgens play a priming role. In support of this notion, exogenous dihydrotestosterone can induce prostatic budding in the UGS of both wild-type female mice and Tfm male mice [23]. Androgen-independent signals are involved in prostate initiation and budding. The WNT signaling pathway is one of the most important signaling pathways playing a dominant role in cell fate determination [24]. The canonical WNT signaling pathway depends on the translocation of the β-catenin protein (encoded by the CTNNB1 gene), and nuclear β-catenin forms a complex with members of the TCF/LEF family to activate the transcription of WNT target genes [25]. The WNT signaling pathway regulates prostate specification and subsequent prostatic budding and epithelial branching morphogenesis as well as prostate stem cell self-renewal. Several WNT ligands and the WNT upstream regulator R-spondin 3 are present in the lower urogenital tract during prostate development and are more abundant in the male UGS than in the female UGS [26]. The β-catenin and WNT/β-catenin-responsive downstream genes Axin2 and Lef1 are highly expressed in the prostatic bud epithelium and colocalize with NKX3-1. Moreover, treatment of UGS explant cultures with a WNT antagonist, such as DKK1, not only decreases the number of prostatic buds but also inhibits NKX3-1 expression, indicating critical roles for WNT/β-catenin during prostate specification and bud formation [27]. Conditional deletion of β-catenin in the E15.5 mouse UGS prevents prostatic differentiation and bud formation. Interestingly, pretreatment of the mouse E15.5 UGS with dihydrotestosterone for 24 h can result in rudimentary bud formation, even after inducing β-catenin deletion by tamoxifen treatment [27]. This indicates that β-catenin is required for initiating prostate differentiation but not for subsequent prostate gland formation. Supporting this conclusion, the specific deletion of β-catenin in adult luminal epithelial cells in the prostate gland in Probasin-Cre mice does not affect glandular homeostasis [28]. However, treatment of cultured postnatal rat ventral prostates with either the WNT agonist WNT3A or WNT antagonist DKK1 leads to a significantly reduced number of branches [29], suggesting a delicate dosing effect of WNT signaling on prostatic epithelial branching morphogenesis. In addition, the noncanonical WNT/calcium pathway, in which noncanonical WNT ligands such as WNT4, WNT5A, and WNT11 mediate the induction of intracellular Ca2+ transients to activate the Ca2+-sensitive kinases CAMK2 and PKC [30], also plays a role in building a unique branch pattern during prostate branching morphogenesis. WNT5A is mainly expressed at the distal tips, and ex vivo experiments show that WNT5A treatment does not affect prostate bud initiation but regulates the size and number of buds [31].

The BMP signaling pathways is necessary for prostate budding/BMP signaling pathway is also critically involved in prostate development. BMP4 is highly expressed in the male UGS from E14 to birth. Exogenous BMP4 can inhibit prostate ductal budding in a dose-dependent manner, and in BMP4 haplo-insufficient adult mice, the prostate contains an increased number of duct tips [32]. These data demonstrate that the BMP signal inhibits prostate ductal budding to ensure an appropriate number of ductal tips for normal prostate development. In addition, activin A is weakly expressed during development, but its expression is upregulated in the prostatic epithelium during puberty. Follistatin and activin receptors are expressed throughout the prostatic epithelium. Functionally, activin A can inhibit prostatic branching in prostate organ cultures, but follistatin, an activin-binding protein that inhibits TGFβ signaling, can increase branching in vitro [33]. Taken together, these data suggest that the TGFβ/BMP signaling pathway negatively regulates prostatic ductal branching morphogenesis.

**Fig. 2:**
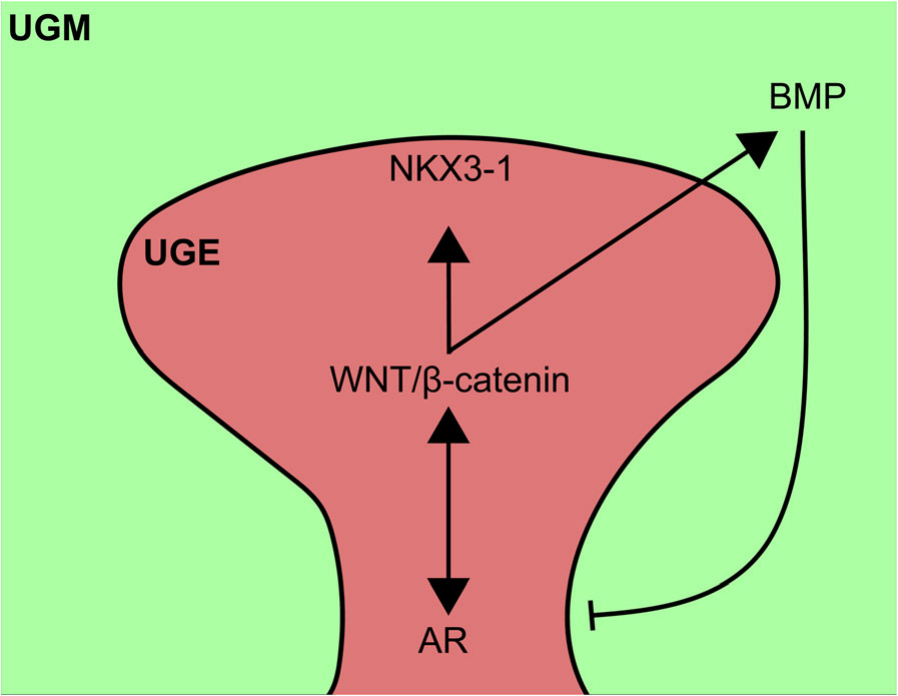
Crosstalk between key signal pathways during prostatic budding. AR signaling pathway activates canonical WNT signaling, which in turn activates the master regulator NKX3-1 to that drives prostatic budding from UGE. In addition, WNT signaling can directly promote AR signal or indirectly inhibit AR signal by activating BMP signal which inhibits AR and prostate ductal budding. AR/WNT/BMP work together to ensure an appropriate number of ductal tips for normal prostate development

BMP signaling pathways synergistically determine prostate development/BMP signaling pathways synergistically determine prostate development (Fig. 2). Conditionally knocking out β-catenin in the UGS results in undetectable NKX3-1 expression, but AR is still highly expressed [28]. These results indicate that prostate lineage specification depends on WNT/β-catenin signaling even when the AR signaling pathway is active. However, after the completion of prostate lineage commitment, prostate development can occur without the canonical WNT signaling pathway [28]. Both ex vivo and tissue implantation experiments demonstrate that when AR is deleted in AXIN2-expressing mouse prostate cells, the formed prostates are relatively small and immature [34]. This indicates the indispensable role for AR in WNT-responsive cells during all the stages of prostate development. In the prostate cancer cell line LNCaP, WNT3A treatment can promote AR binding to the promoter regions of WNT target genes such as MYC and CYCLIN D1; additionally, AR and β-catenin can be recruited to the promoter and enhancer regions of the AR target gene PSA [35]. Another report also noted that WNT/β-catenin could increase AR expression through the binding of LEF1 to the promotor of AR [36]. In addition, active WNT/β-catenin can activate BMP signaling in prostatic bud tips to inhibit inappropriate prostatic budding and together ensure the initiation of prostate development [37]. β-catenin transcriptionally upregulates the expression of TGFβ2, TGFβ3, and BMP4 in prostate stromal cells, and the activated TGFβ pathway suppresses basal cell proliferation [37, 38]. The TGFβ and AR signaling pathways in the stroma can affect the WNT signaling pathway, which helps to limit prostatic regression [39]. Therefore, a balance between the WNT and TGFβ/BMP signaling pathways is necessary for prostate budding.

## Prostate lineage differentiation from pluripotent stem cells

**Fig. 3:**
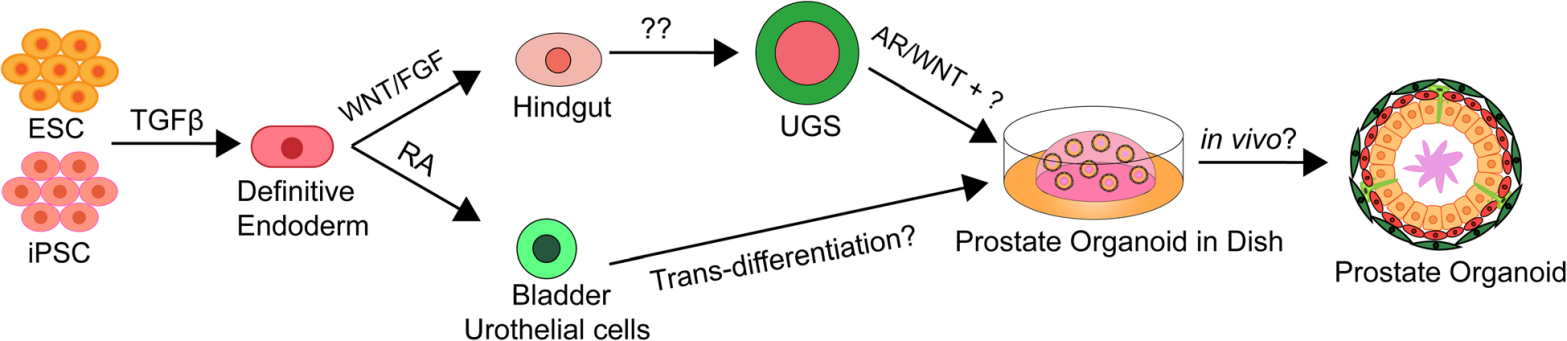
Summary and proposal of prostate differentiation from human pluripotent stem cells. Human ESC/iPSC can respond to TGFβ signaling pathway to differentiate into definitive endoderm, from which there are two paths to generate prostate organoids: mimic the prostate development process (from definitive endoderm to hindgut stage by WNT and FGF signaling pathways, then to UGS by certain signals, and eventually to prostate organoids by AR and other signaling pathways; or direct to bladder urothelial cells by RA, and then transdifferentiate into prostate organoids

Current knowledge on prostate development is mainly derived from rodent animal models and prostate cancer cell lines; however, there are significant differences between the human prostate and rodent prostate, such as differences in histology and morphology. Limited by the shortage of human prostate materials, especially embryonic specimens, a more widespread method is urgently required. Human pluripotent stem cells, including embryonic stem cells (ESCs) and induced pluripotent stem cells (iPSCs), harbor the capacity to undergo multilineage differentiation to generate almost all cell types composing the body. Therefore, human pluripotent stem cells may provide a promising source to study human prostate development (Fig. 3). As early as 2006, a study by Taylor et al. showed that human ESCs are able to differentiate into the prostatic lineage by utilizing a co-transplantation assay [40]. They constructed a hetero-species recombinant tissue composed of the mouse UGM or rat seminal vesicle mesenchyme and human ESCs under the renal capsule of immunodeficient mice and observed prostate-like tissue within 8–12 weeks. Key transcription factors such as AR and NKX3-1 and the epithelial cell markers P63, CK8, and CK18 were detected at 4 weeks, while a mature prostate that expressed prostate-specific antigen (PSA) and exhibited prostate layer structures appeared 8–12 weeks after grafting [40]. However, this approach utilized co-culture and transplantation and was inefficient, hindering further mechanistic study and applications.

Prostate organogenesis is a stepwise process. The prostate arises from the UGS, which is a caudal extension of the endoderm-derived hindgut [1]. It was demonstrated that definitive endoderm specification is mainly controlled by the NODAL/SMAD signaling pathway [41]. Consistently, human ESCs can be efficiently differentiated into the definitive endoderm lineage upon activin A treatment in low-serum cultures [42]. The endoderm germ layer is an undetermined sheet of cells, it forms a primitive tube and becomes regionally specified along the anterior-posterior axis later, and FGF signaling is necessary for establishing the posterior endoderm [43]. In addition, high β-catenin activity is observed in the posterior endoderm and inhibits the foregut fate [44]. Therefore, agonists of FGF and WNT signals are used to differentiate the definitive endoderm into CDX2-positive hindgut lineages [45].

Although many achievements have been made in efficiently differentiating human ESCs/iPSCs into the definitive endoderm and hindgut, there are very few reports on the directed differentiation from the hindgut to the UGS and subsequently to the prostate lineage. The master signals of this process are still largely unknown, but some attempts have been made to elucidate this process. Inspired by the early success achieved in generating prostate lineage cells following co-transplantation of human ESCs and the rodent UGM [40], Hepburn and colleagues first generated prostate-derived human iPSCs and differentiated iPSCs into the definitive endoderm. Then, the differentiated endoderm cells were cocultured with the UGM in vitro. They observed increased efficiency of prostatic epithelial differentiation [46]. However, there are still many problems in directed prostatic differentiation requiring further exploration.

stem cells are capable of generating a range of differentiated/stem cells are capable of generating a range of differentiated cells, including prostate lineages [3]. A tissue recombination experiment using the UGM with adult bladder epithelia suggests that the UGM can induce bladder epithelial cells to form a prostate through a trans-differentiation mechanism that requires stromal TGF-β signaling to mediate epithelial WNT activity [47]. In addition, bladder urothelium differentiation from human ESC-derived endoderm has been successfully achieved in vitro. Kang and colleagues first generated bladder urothelial cells from human ESCs under serum- and feeder-free conditions with retinoic acid, and the bladder urothelial cells highly expressed urothelium-specific genes such as UPIB, UPII, UPIIIA, P63, and CK7 [48]. This may represent an alternative approach to guide prostate generation from human ESCs/iPSCs (Fig. 3).

## Prostate organoids

An organoid is an advanced technology that mimics the hallmarks, cell types, and even the structure and functions of real organs and hence provides an alternative in vitro model for developmental study and disease modeling [49]. ESCs/iPSCs provide an ideal source to construct prostate organoids. Human ESC/iPSC-derived organoids can be used as powerful platforms in modeling human organ development and disease. For example, human gastric organoids have been generated in vitro from pluripotent stem cells through manipulation of the FGF, WNT, BMP, retinoic acid, and EGF signaling pathways as well as three-dimensional culture. Generated gastric organoids have been used to identify novel signals that regulate early endoderm patterning or to study the function of the transcription factor NEUROG3 in gastric endocrine cell differentiation. Moreover, human gastric organoids have been used to mimic the pathophysiological response of the gastric epithelium to *H. pylori*, which manifests the potential of these organoids in drug discovery and modeling the early stages of gastric disease and even cancer [50]. Similar achievements have been made in studying human lung development during gestation, recapitulating fibrotic lung disease in vitro by introducing the mutation in HPS1 that causes an early-onset form of intractable pulmonary fibrosis [51] and modeling colonic familial adenomatous polyposis, which identified geneticin as a promising drug for APC-mutant patients [52]. 

**Fig. 4:**
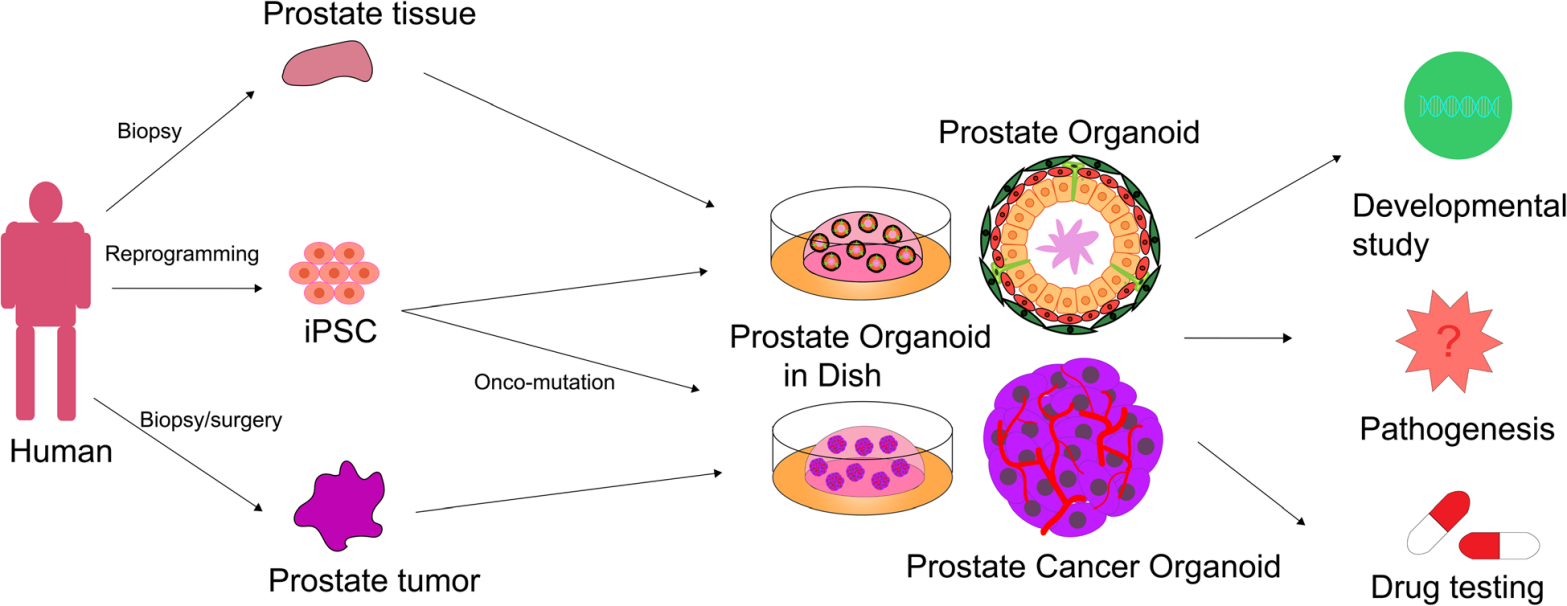
Application overview of human prostate organoid technology. Prostate organoids can be generated from prostate primary tissues or tumor samples directly or derived from iPSC through somatic reprogramming, differentiation, and co-culture technology. Prostate organoids have wide application potentials and provide a valuable resource to study human development, model pathogenesis, and test drugs

Prostate organoids can be derived from pluripotent stem cells, prostatic progenitor cells, or primary prostate biopsy samples (Fig. 4). Based on the strategy that the R-spondin 1-based WNT-activation culture method allows long-term propagation of murine and human prostate epithelium, both basal and luminal populations have been demonstrated to contain bipotent progenitor cells, therefore making it possible to establish murine and human prostate organoids in vitro. Basal- or luminal-derived prostate organoids express AR, NKX3-1, and prostate epithelium layer markers including P63, CK5, and CK8 [53]. Moreover, those organoids exhibit testosterone responsiveness upon dihydrotestosterone addition or withdrawal. Also, the Zeb1+ prostate epithelial cells are multipotent prostate basal stem cells that can self-renew and therefore capable of generating functional prostate organoids at the single-cell level [54]. Prostate organoids derived from iPSCs were successfully generated using a co-culture technique with the urogenital sinus mesenchyme, and early prostate organoids can be generated within several weeks [46].

Organoids can be used to study the functions of genes involved in prostate cancer initiation by gene editing [55]. Genetic ablation studies reveal the indispensable role of Zeb1 in prostate development [54]. In addition to normal prostate cells, a testosterone-responsive prostate organoid culture system derived from advanced prostate cancer tissue was developed to study prostate homeostasis and tumorigenesis [56, 57]. Recent research has demonstrated that prostate stromal cells can increase organoid formation efficiency and influence organoid branching morphogenesis via cell-cell contact and secrete soluble growth factors that may regulate branching [58]. This system provides a powerful model to study the functions of developmental regulators or oncogenes, such as MYC and AKT1 [55]. The high-throughput model can rapidly generate human prostate tissue ex vivo and in vitro, which makes it a better model for studying prostate development and disease than slow, inefficient, and laborious prostate organoids derived from primary cultures [46] (Fig. 4).

## Conclusion

In this review, we have briefly introduced prostate development and summarized the major signaling pathways involved in prostate development and differentiation (i.e., the AR, WNT, and TGF-β/BMP signaling pathways). To advance our understandings of human prostate development and prostatic disease, prostate organoids based on human pluripotent stem cells would be a promising and valuable tool with some challenges.

## Data Availability

All data analyzed in this study are included in this published article.

## Abbreviations

AR: Androgen receptor
UGS: Urogenital sinus
UGE: Urogenital sinus epithelium
UGM: Urogenital sinus mesenchyme
BRAW: Branching and annihilating random walks
Tfm: Testicular feminization mutant
ESC: Embryonic stem cell
iPSC: Induced pluripotent stem cell

## References

[1] Seifert AW, Harfe BD, Cohn MJ (2008). Cell lineage analysis demonstrates an endodermal origin of the distal urethra and perineum. Dev Biol.

[2] Cunha GR, Fujii H, Neubauer BL, Shannon JM, Sawyer L, Reese BA (1983). Epithelial-mesenchymal interactions in prostatic development. I. Morphological observations of prostatic induction by urogenital sinus mesenchyme in epithelium of the adult rodent urinary bladder. J Cell Biol.

[3] Staack A, Hayward SW, Baskin LS, Cunha GR (2005). Molecular, cellular and developmental biology of urothelium as a basis of bladder regeneration. Differentiation..

[4] Feldman SC, Bloch E (1978). Developmental pattern of testosterone synthesis by fetal rat testes in response to luteinizing hormone. Endocrinology..

[5] Berman DM, Tian H, Russell DW (1995). Expression and regulation of steroid 5 alpha-reductase in the urogenital tract of the fetal rat. Mol Endocrinol.

[6] Lasnitzki I, Mizuno T (1980). Prostatic induction: interaction of epithelium and mesenchyme from normal wild-type mice and androgen-insensitive mice with testicular feminization. J Endocrinol.

[7] Kellokumpu-Lehtinen P, Santti R, Pelliniemi LJ (1980). Correlation of early cytodifferentiation of the human fetal prostate and Leydig cells. Anat Rec.

[8] Cunha GR, Donjacour AA, Cooke PS, Mee S, Bigsby RM, Higgins SJ (1987). The endocrinology and developmental biology of the prostate. Endocr Rev.

[9] Gao N, Zhang J, Rao MA, Case TC, Mirosevich J, Wang Y (2003). The role of hepatocyte nuclear factor-3 alpha (Forkhead Box A1) and androgen receptor in transcriptional regulation of prostatic genes. Mol Endocrinol.

[10] Zhao JC, Fong KW, Jin HJ, Yang YA, Kim J, Yu J (2016). FOXA1 acts upstream of GATA2 and AR in hormonal regulation of gene expression. Oncogene..

[11] Adams EJ, Karthaus WR, Hoover E, Liu D, Gruet A, Zhang Z (2019). FOXA1 mutations alter pioneering activity, differentiation and prostate cancer phenotypes. Nature..

[12] Bhatia-Gaur R, Donjacour AA, Sciavolino PJ, Kim M, Desai N, Young P (1999). Roles for Nkx3.1 in prostate development and cancer. Genes Dev.

[13] Talos F, Mitrofanova A, Bergren SK, Califano A, Shen MM (2017). A computational systems approach identifies synergistic specification genes that facilitate lineage conversion to prostate tissue. Nat Commun.

[14] Toivanen R, Shen MM (2017). Prostate organogenesis: tissue induction, hormonal regulation and cell type specification. Development..

[15] Cunha GR, Vezina CM, Isaacson D, Ricke WA, Timms BG, Cao M (2018). Development of the human prostate. Differentiation..

[16] Hannezo E, Scheele C, Moad M, Drogo N, Heer R, Sampogna RV (2017). A unifying theory of branching morphogenesis. Cell..

[17] Huang L, Pu Y, Alam S, Birch L, Prins GS (2005). The role of Fgf10 signaling in branching morphogenesis and gene expression of the rat prostate gland: lobe-specific suppression by neonatal estrogens. Dev Biol.

[18] Ghosh S, Lau H, Simons BW, Powell JD, Meyers DJ, De Marzo AM (2011). PI3K/mTOR signaling regulates prostatic branching morphogenesis. Dev Biol.

[19] Cutress ML, Whitaker HC, Mills IG, Stewart M, Neal DE (2008). Structural basis for the nuclear import of the human androgen receptor. J Cell Sci.

[20] Ekman P (2000). The prostate as an endocrine organ: androgens and estrogens. Prostate Suppl.

[21] Quigley CA, De Bellis A, Marschke KB, el-Awady MK, Wilson EM, French FS (1995). Androgen receptor defects: historical, clinical, and molecular perspectives. Endocr Rev.

[22] Lai KP, Yamashita S, Vitkus S, Shyr CR, Yeh S, Chang C (2012). Suppressed prostate epithelial development with impaired branching morphogenesis in mice lacking stromal fibromuscular androgen receptor. Mol Endocrinol.

[23] Allgeier SH, Lin TM, Moore RW, Vezina CM, Abler LL, Peterson RE (2010). Androgenic regulation of ventral epithelial bud number and pattern in mouse urogenital sinus. Dev Dyn.

[24] Logan CY, Nusse R (2004). The Wnt signaling pathway in development and disease. Annu Rev Cell Dev Biol.

[25] Li VS, Ng SS, Boersema PJ, Low TY, Karthaus WR, Gerlach JP (2012). Wnt signaling through inhibition of beta-catenin degradation in an intact Axin1 complex. Cell..

[26] Mehta V, Abler LL, Keil KP, Schmitz CT, Joshi PS, Vezina CM (2011). Atlas of Wnt and R-spondin gene expression in the developing male mouse lower urogenital tract. Dev Dyn.

[27] Kruithof-de Julio M, Shibata M, Desai N, Reynon M, Halili MV, Hu YP (2013). Canonical Wnt signaling regulates Nkx3.1 expression and luminal epithelial differentiation during prostate organogenesis. Dev Dyn.

[28] Simons BW, Hurley PJ, Huang Z, Ross AE, Miller R, Marchionni L (2012). Wnt signaling though beta-catenin is required for prostate lineage specification. Dev Biol.

[29] Wang BE, Wang XD, Ernst JA, Polakis P, Gao WQ (2008). Regulation of epithelial branching morphogenesis and cancer cell growth of the prostate by Wnt signaling. PLoS One.

[30] Veeman MT, Axelrod JD, Moon RT (2003). A second canon. Functions and mechanisms of beta-catenin-independent Wnt signaling. Dev Cell.

[31] Huang L, Pu Y, Hu WY, Birch L, Luccio-Camelo D, Yamaguchi T (2009). The role of Wnt5a in prostate gland development. Dev Biol.

[32] Lamm ML, Podlasek CA, Barnett DH, Lee J, Clemens JQ, Hebner CM (2001). Mesenchymal factor bone morphogenetic protein 4 restricts ductal budding and branching morphogenesis in the developing prostate. Dev Biol.

[33] Cancilla B, Jarred RA, Wang H, Mellor SL, Cunha GR, Risbridger GP (2001). Regulation of prostate branching morphogenesis by activin A and follistatin. Dev Biol.

[34] He Y, Hooker E, Yu EJ, Wu H, Cunha GR, Sun Z (2018). An indispensable role of androgen receptor in Wnt responsive cells during prostate development, maturation, and regeneration. Stem Cells.

[35] Schweizer L, Rizzo CA, Spires TE, Platero JS, Wu Q, Lin TA (2008). The androgen receptor can signal through Wnt/beta-catenin in prostate cancer cells as an adaptation mechanism to castration levels of androgens. BMC Cell Biol.

[36] Li Y, Wang L, Zhang M, Melamed J, Liu X, Reiter R (2009). LEF1 in androgen-independent prostate cancer: regulation of androgen receptor expression, prostate cancer growth, and invasion. Cancer Res.

[37] Mehta V, Schmitz CT, Keil KP, Joshi PS, Abler LL, Lin TM (2013). Beta-catenin (CTNNB1) induces Bmp expression in urogenital sinus epithelium and participates in prostatic bud initiation and patterning. Dev Biol.

[38] Wei X, Zhang L, Zhou Z, Kwon OJ, Zhang Y, Nguyen H (2019). Spatially restricted stromal Wnt signaling restrains prostate epithelial progenitor growth through direct and indirect mechanisms. Cell Stem Cell.

[39] Placencio VR, Sharif-Afshar AR, Li X, Huang H, Uwamariya C, Neilson EG (2008). Stromal transforming growth factor-beta signaling mediates prostatic response to androgen ablation by paracrine Wnt activity. Cancer Res.

[40] Taylor RA, Cowin PA, Cunha GR, Pera M, Trounson AO, Pedersen J (2006). Formation of human prostate tissue from embryonic stem cells. Nat Methods.

[41] Vincent SD, Dunn NR, Hayashi S, Norris DP, Robertson EJ (2003). Cell fate decisions within the mouse organizer are governed by graded Nodal signals. Genes Dev.

[42] D'Amour KA, Agulnick AD, Eliazer S, Kelly OG, Kroon E, Baetge EE (2005). Efficient differentiation of human embryonic stem cells to definitive endoderm. Nat Biotechnol.

[43] Dessimoz J, Opoka R, Kordich JJ, Grapin-Botton A, Wells JM (2006). FGF signaling is necessary for establishing gut tube domains along the anterior-posterior axis in vivo. Mech Dev.

[44] McLin VA, Rankin SA, Zorn AM (2007). Repression of Wnt/beta-catenin signaling in the anterior endoderm is essential for liver and pancreas development. Development..

[45] Spence JR, Mayhew CN, Rankin SA, Kuhar MF, Vallance JE, Tolle K (2011). Directed differentiation of human pluripotent stem cells into intestinal tissue in vitro. Nature..

[46] Hepburn AC, Curry EL, Moad M, Steele RE, Franco OE, Wilson L, et al. Propagation of Human Prostate Tissue From Induced Pluripotent Stem Cells. Stem Cells Transl Med. 2020;9(7):734-45. 10.1002/sctm.19-0286 PMC7308643 32170918

[47] Li X, Wang Y, Sharif-Afshar AR, Uwamariya C, Yi A, Ishii K (2009). Urothelial transdifferentiation to prostate epithelia is mediated by paracrine TGF-beta signaling. Differentiation..

[48] Kang M, Kim HH, Han YM (2014). Generation of bladder urothelium from human pluripotent stem cells under chemically defined serum- and feeder-free system. Int J Mol Sci.

[49] Clevers H (2016). Modeling development and disease with Organoids. Cell..

[50] McCracken KW, Cata EM, Crawford CM, Sinagoga KL, Schumacher M, Rockich BE (2014). Modelling human development and disease in pluripotent stem-cell-derived gastric organoids. Nature..

[51] Chen YW, Huang SX, de Carvalho A, Ho SH, Islam MN, Volpi S (2017). A three-dimensional model of human lung development and disease from pluripotent stem cells. Nat Cell Biol.

[52] Crespo M, Vilar E, Tsai S-Y, Chang K, Amin S, Srinivasan T (2017). Colonic organoids derived from human induced pluripotent stem cells for modeling colorectal cancer and drug testing. Nat Med.

[53] Karthaus WR, Iaquinta PJ, Drost J, Gracanin A, van Boxtel R, Wongvipat J (2014). Identification of multipotent luminal progenitor cells in human prostate organoid cultures. Cell..

[54] Wang X, Xu H, Cheng C, Ji Z, Zhao H, Sheng Y (2020). Identification of a Zeb1 expressing basal stem cell subpopulation in the prostate. Nat Commun.

[55] Park JW, Lee JK, Phillips JW, Huang P, Cheng D, Huang J (2016). Prostate epithelial cell of origin determines cancer differentiation state in an organoid transformation assay. Proc Natl Acad Sci U S A.

[56] Beshiri ML, Tice CM, Tran C, Nguyen HM, Sowalsky AG, Agarwal S (2018). A PDX/organoid biobank of advanced prostate cancers captures genomic and phenotypic heterogeneity for disease modeling and therapeutic screening. Clin Cancer Res.

[57] Puca L, Bareja R, Prandi D, Shaw R, Benelli M, Karthaus WR (2018). Patient derived organoids to model rare prostate cancer phenotypes. Nat Commun.

[58] Richards Z, McCray T, Marsili J, Zenner ML, Manlucu JT, Garcia J (2019). Prostate stroma increases the viability and maintains the branching phenotype of human prostate organoids. iScience.

